# Identifying Prescription-Opioid-Related Risks Using Prescription Drug Monitoring Programs’ Algorithms and Clinical Screening Tools

**DOI:** 10.3390/pharmacy11050164

**Published:** 2023-10-13

**Authors:** Louisa Picco, Monica Jung, Helena Cangadis-Douglass, Tina Lam, Suzanne Nielsen

**Affiliations:** 1 Monash Addiction Research Centre, Peninsula Campus, Monash University, 47-49 Moorooduc Hwy Frankston, Victoria 3199, Australia; monica.jung@monash.edu (M.J.); helena.cangadis-douglass1@monash.edu (H.C.-D.); tina.lam@monash.edu (T.L.);; 2 Centre for Medicine Use and Safety (CMUS), Parkville Campus, Monash University, 381 Royal Parade Parkville, Victoria 3052, Australia

**Keywords:** prescription drug monitoring program, prescription-opioid-related risks, clinical screening tool, community pharmacists

## Abstract

Background: Pharmacists adopt various approaches to identifying prescription-opioid-related risks and harms, including prescription drug monitoring programs (PDMPs) and clinical screening tools. This study aims to compare ‘at-risk’ patients according to the published Australian PDMP algorithms with the validated Routine Opioid Outcome Monitoring (ROOM) clinical screening tool. Methods: Data were used from an implementation study amongst people who had been prescribed regular opioids. We examined the results from ROOM and the patients’ dispensing history over the previous 90 days. A chi-squared test was used to examine the association between risk according to (i) a PDMP alert and a clinical risk per ROOM; (ii) a PDMP alert and positive screening for opioid use disorder; and (iii) a PDMP ‘high-dose’ alert (average of >100 mg OME/day in the past 90 days) and any ROOM-validated risk. Results: No significant associations were found between being ‘at-risk’ according to any of the PDMP alerts and clinical risk as identified via the ROOM tool (x^2^ = 0.094, *p* = 0.759). There was only minimal overlap between those identified as ‘at-risk’ via PDMP alerts and those meeting the clinical risk indicators; most patients who were ‘at-risk’ of clinical opioid-related risk factors were not identified as ‘at-risk’ based on PDMP alerts. Conclusions: PDMP alerts were not predictive of clinical risk (as per the ROOM tool), as many people with well-established clinical risks would not receive a PDMP alert. Pharmacists should be aware that PDMPs are limited to identifying medication-related risks which are derived using algorithms; therefore, augmenting PDMP information with clinical screening tools can help create a more detailed narrative of patients’ opioid-related risks.

## 1. Introduction

Opioid prescriptions for chronic, non-cancer pain have increased substantially in recent decades, despite insufficient evidence supporting long-term opioid use for this indication [1]. The long-term and/or non-medical use of prescription opioids is associated with significant harm and, consequently, many high-income countries are grappling with an opioid-related public health crisis [2,3]. The US has the highest consumption of prescription opioids, far exceeding that of other countries, and has reported over 80,000 opioid-related overdose deaths in 2021 alone [4]. In Australia, opioids are the most common drug type associated with drug-induced deaths, resulting in 1091 deaths in 2020, of which nearly two-thirds involved prescription opioids [5]. Overdoses can also place considerable strain on the healthcare system, including ambulances, emergency departments (EDs), and other hospital modalities.

In addition to fatal and non-fatal overdoses, long-term prescription opioid use is also associated with a range of other harms. Some of the most common harms include (i) tolerance, physical dependence, and withdrawal; (ii) non-medical use and associated behaviours; and (iii) prescription opioid dependence and opioid use disorder (OUD). Collectively, these harms result in substantial health, social, and economic costs and burdens [6].

In order to mitigate the risks and reduce the harms associated with long-term prescription opioid use, a range of policies and strategies have been adopted, many of which are implemented simultaneously. These strategies commonly adopt different approaches, which often fall under the umbrella term of ‘harm minimisation’ and include three main approaches. These include the following: (i) demand reduction, which is designed to reduce drug use; (ii) supply reduction, which aims to reduce the supply of drugs; and (iii) harm reduction, which aims to reduce the negative effects of health behaviours without aiming to reduce drug use per se [7]. Healthcare providers, therefore, must balance the safe and effective supply of opioids for pain management with mitigating the risks associated with opioid use. 

More specifically, pharmacists are at the clinical interface between prescribers and patients, and are ideally positioned to identify and respond to prescription-opioid-related risks and harms. However, pharmacists are often an underutilised resource and their role in reducing prescription-opioid-related risks has been neglected [8]. They are also the most easily accessed healthcare provider in Australia, providing more free advice to patients than any other healthcare provider [9]. Therefore, the role of pharmacists can be enhanced to improve patient care, identify and respond to risks, and avert medication-related harms [8,10]. 

To do this, they can adopt various approaches, one of the most common being the use of prescription drug monitoring programs (PDMPs). PDMPs are electronic databases that collate patient-level prescribing and dispensing information for ‘high-risk’ medications including opioids [11] and are designed to aid healthcare providers’ decision making, facilitate earlier intervention, and reduce diversion and related harms [12]. PDMPs have evolved significantly, largely due to advances in technology. For example, more recently, PDMPs have been developed to capture information in real-time, as monitored medications are prescribed and dispensed. 

PDMPs use an algorithm to generate alerts, indicating potentially ‘high-risk’ scenarios such as ‘multiple prescribers’ episodes and high-dose and high-risk drug combinations [13]. Currently, though, there is a limited evidence base to support such criteria for generating alerts [14]. PDMP use has also been associated with a range of unintended consequences, including the abrupt cessation of prescribed opioids, the over-reliance on automated alerts, stigma, and even increases in heroin-related overdoses [15,16,17]. PDMPs are a state-based policy, and, therefore, there are often differences both within and across countries. These differences commonly relate to the risks PDMPs capture, the medications they monitor, and whether there are mandates related to their registration and use, as well as the types of information captured within the PDMP. Such differences pose additional challenges, as it is difficult to elucidate specific nuances or features that may contribute to expected and unexpected or unintended outcomes. 

Another strategy to help identify at-risk patients is the use of screening tools. Existing tools such as the Opioid Risk Tool [18], the Current Opioid Misuse Measure [19], and Opioid-Related Behaviours In Treatment [20] are limited by their sole focus on dependence or aberrant behaviours and are not designed for or specifically validated among people who are prescribed opioids. Other key risk factors that are not measured using these existing tools include unmanaged pain, mood disorders, and dependence or addiction [21,22,23]. The Routine Opioid Outcome Monitoring (ROOM) tool measures these clinical domains and was developed specifically for use in primary care and pharmacy settings. The ROOM tool incorporates the ‘4As’ model of monitoring opioid outcomes. These comprise analgesia, activity (e.g., psychosocial functioning), adverse effects, and aberrant drug-related behaviours [24], as well as the domains of affect (mood) and risky alcohol use. This validated patient-administered tool [25] screens for opioid-related clinical risk outcomes and comprises brief measures of pain functioning [26], prescription opioid use disorder (OUD) [27], depression [28], risky alcohol use [29], and constipation [30]. 

Adopting evidence-based policies and approaches that not only identify a range of risks but also improve opioid safety and patient outcomes is essential [31], and the pharmacists’ role in this risk identification process has increased substantially in recent years [32,33]. Best practice recommends that all patients who are prescribed opioids should have their opioid-related risks and outcomes monitored using a ‘universal precautions’ approach [34]. The ways in which pharmacists identify prescription-opioid-related risks can vary and may comprise one or a combination of approaches. Irrespective of the approach, the emphasis should be on improving patient outcomes and safety, recognising that no single approach can adequately identify all possible risks [35]. It is currently unclear if different approaches identify the same patients to be at-risk or whether some patients can be identified as at-risk through one approach but not meet the risk criteria according to another. The current study sought to address this gap and aimed to compare patients identified as having prescription-opioid-related risks through two common approaches: PDMP alerts and validated screening tools. 

## 2. Materials and Methods

### 2.1. Participants and Procedures

Dispensing history from a pilot implementation study using the ROOM tool [36] was used (UNSW HREC Reference: #HC17760). Sixty-four pharmacists from 23 community pharmacies in Victoria and New South Wales, Australia, were recruited in 2019. Eligibility criteria for patients included: (1) receiving a repeat supply of opioids for non-cancer pain; (2) being aged ≥ 18 years; (3) the ability to provide voluntary informed consent; and (4) being willing and able to self-complete the ROOM tool in the pharmacy. Cross-sectional data were collected for 152 patients as part of the original implementation study. 

The inclusion criteria for the current study required patients to be prescribed regular opioids, which was defined as ≤60 days between opioid prescriptions [37]. Thirty-three participants were excluded from the current analysis due to irregular prescribed opioid use and therefore would be unlikely to be the target audience for opioid policies designed to reduce opioid-related risks such as PDMPs.

Patient demographic information collected included age and gender. Dispensing history data for all medications prescribed over the 90 days before participation were extracted. Medications of interest for the current analysis included any opioids, benzodiazepines, zolpidem, and zopiclone, as these are all medications monitored by Victoria’s PDMP. 

Responses from the 12-item patient-completed online ROOM tool were used to identify patients who met established cut-offs for severe pain (despite current opioid use), prescription OUD, depression, and risky alcohol use [25]. Pain was assessed using the ‘PEG’, which measures pain intensity, interference with enjoyment of life, and interference with general activity on a 0–10 scale, where mean scores for the three items were dichotomised and a score of ≥7 represented “severe pain” [38]. Prescription OUD was assessed by the OWLS, a validated tool that measures symptom risk with four aspects of prescription opioid use: overuse, worrying, losing interest, and feeling slowed down, sluggish, or sedated [27]. A total score of three or more over the four items indicated likely symptoms of prescription OUD. Depression was measured using the PHQ-2, where a cut-off of 3 indicated current symptomatic depression [28]. Risky alcohol use was measured using a single screening question which was: “How many times in the past year have you had X or more drinks in a day?” (where X is five for men and four for women) and a response of ≥1 is considered positive for risky alcohol use [29].

### 2.2. ‘At-Risk’ Definitions

Dispensing histories relating to patients’ medication use on the day they completed the ROOM tool were used to determine which patients would receive published Victorian PDMP algorithm-generated alerts, identifying patients ‘at-risk’ related to high doses or high-risk drug combinations.

Firstly, the Oral Morphine Equivalent (OME) dose was calculated to identify which patients would receive a high dose alert [39]. Two authors (MJ and HCD) calculated the OME [39] for all opioids prescribed during the 90 days before study participation, then summed and divided this by 90 to provide an average OME per day. This was used to indicate a PDMP alert for high- (>100 mg OME) and medium-dose risk (50–100 mg OME). A cross-check on 15% of all OME calculations was undertaken to ensure accuracy. 

Secondly, medication data relevant to the PDMP high-risk drug combination alert were extracted. This alert occurs when a patient is prescribed specific medication combinations including (i) methadone and benzodiazepines, (ii) methadone and long-acting opioids, (iii) fentanyl and benzodiazepines, and (iv) fentanyl and long-acting opioids. 

The definition of ‘long-acting opioids’ varied depending on the medication combinations. For (ii) “methadone and long-acting opioids”, it referred to slow-release or modified-release opioids, buprenorphine patches, fentanyl patches, and opioid injections. For (iv) “fentanyl and long-acting opioids”, it comprised slow-release or modified-release opioids, buprenorphine patches, buprenorphine oral and injectable formulations used for the treatment of opioid dependence, and opioid injections; however, it excluded methadone. Any PDMP alert was defined as patients who would receive either of the two dose alerts (i.e., high dose of >100 mg OME or medium dose of 50–100 mg OME) or the high-risk drug combination alert.

The ROOM tool defined ‘any clinical risk’ as meeting criteria for any one of the four risk indicators of severe pain (despite opioid use), prescription OUD, depression, or risky alcohol use. Whilst the ROOM clinical screening tool also captures data related to constipation, this was not considered a risk factor for this analysis, although it is considered an important side effect that warrants monitoring.

### 2.3. Statistical Analysis

Descriptive statistics were used to summarise the sample characteristics. The chi-squared test was used to examine the association between various risks identified via the Victorian PDMP alerts and the ROOM clinical screening tool. More specifically, we explored whether there was an association between: (i) any PDMP alert and any ROOM risk indicator; (ii) any PDMP alert and prescription OUD only (i.e., one of the ROOM risks), and (iii) PDMP high-dose alert (average of >100 mg OME/day in the past 90 days) and any ROOM risk indicators. All statistical analyses were conducted using SPSS Version 25.

## 3. Results

### 3.1. Sample Characteristics

The sample comprised 119 patients prescribed regular opioids (less than 60 days between opioid prescriptions). Just over half of the sample were female (*n* = 66, 55.5%), and aged 64 or younger (*n* = 65, 54.6%; Table 1). One in three (*n* = 40) was prescribed benzodiazepines or z-drugs (e.g., zolpidem and zopiclone), which, in addition to opioids, are also medications monitored by the PDMP. Polypharmacy, which was defined as being prescribed five or more medications, was common amongst the sample (*n* = 70, 59%).

### 3.2. Any PDMP Alert and Any ROOM Clinical Risk Factor

The majority of the sample had at least one clinical risk identified by the ROOM tool (*n* = 94, 79%), with 55 meeting the criteria for two or more. Thirteen participants met the criteria for three of the four ROOM risks, while six participants met the criteria for all four ROOM risk indicators. The most common ROOM risk indicator was severe pain (*n* = 65, 69.1%), followed by risky alcohol use (*n* = 45, 47.9%) and prescription OUD (*n* = 37, 39.4%), while 27 (28.7%) met the criteria for depression. 

Being at-risk according to the PDMP alerts was less common. In total, 46 (38.7%) participants met the criteria for any PDMP alert, of which the majority (*n* = 44) related to the dose alerts. More specifically, 16 would receive a high-dose (>100 mg OME) PDMP alert, while 28 would receive a medium-dose PDMP alert (50–100 OME; *n* = 28). Five participants met the criteria for the high-risk drug-combination-based alert, and three participants met the criteria for both a dose-related and high-risk drug combination alert (Table 1, Supplementary Figure S1). Thirty-seven participants were ‘at-risk’ according to any PDMP alert and any ROOM risk, with no significant association between the two tools (x^2^ = 0.094, *p* = 0.759) (Table 2). 

### 3.3. Any PDMP Alert and Prescription Opioid Use Disorder

Forty-six participants were identified as ‘at-risk’ based on any PDMP alerts (i.e., they would receive either of the dose alerts (high dose of >100 mg OME or medium dose of 50–100 mg OME) or the high-risk drug combination alert). Thirty-seven participants were ‘at-risk’ of prescription OUD (as assessed by the OWLS prescription OUD tool, within the ROOM tool) and twelve participants were identified as ‘at-risk’ by both tools. There was no significant association between being ‘at-risk’ for prescription OUD and receiving a PDMP alert (x^2^ = 0.877, *p* = 0.349) (Table 2).

### 3.4. High-Dose PDMP Alert and Any ROOM Clinical Risk

There was no association between receiving a high-dose (>100 mg OME) PDMP alert and any ROOM risk (x^2^ = 0.057, *p* = 0.812, Table 2). In total, 94 participants met the criteria for any ROOM risk indicators, 16 participants elicited a PDMP high-dose alert (>100 mg OME), and 13 were ‘at-risk’ according to both tools, with the most common ROOM risks being severe pain (despite opioid use) (*n* = 12) and depression (*n* = 6). 

## 4. Discussion

The current study compared patients identified as having prescription-opioid-related risks according to published Victorian PDMP algorithms and two clinical screening tools (namely the ROOM and OWLS), amongst a sample of people prescribed regular opioids. The results revealed that PDMP alerts and clinical screening tools identified different patients as being at-risk, with minimal overlap, and no significant association between these risk identification approaches. These findings have a range of implications as these two approaches are adopted in pharmacy settings.

PDMP alerts were not found to be predictive of clinical risk (as assessed by the ROOM tool). In fact, many people with well-established clinical risks would not receive a PDMP alert, including those that met the prescription OUD criteria, a group who are at higher risk of overdose, and other opioid-related harms [40]. Furthermore, PDMP high-dose alerts are commonly used to identify ‘at-risk’ populations; however, research has found the majority of this risk is explained by other clinical factors. For example, a five-year, prospective cohort study of people prescribed opioids for chronic non-cancer pain found that those on high doses were much more likely to also have pre-existing severe pain, mental health co-morbidities including depression, and substance use disorders, and it is these pre-existing factors that may influence the dose [41]. Severe pain is a significant risk factor for prescription opioid misuse [22] and was the most common ROOM risk indicator amongst the current sample, and amongst those identified as ‘at-risk’ based on the PDMP high-dose alert. Half of those who met the PDMP high-dose alert criteria were also ‘at-risk’ of depression, another well-known risk factor for non-medical use [42], and this is also associated with overdose risk [43]. Given the clinical nature of depression, it is important that healthcare providers can identify this risk and then carefully manage it, alongside other possible prescription-opioid-related risks. 

Given this lack of association between these two risk identification approaches, pharmacists should be cognisant that PDMP alerts may not be effective at identifying patients with clinical risks and are limited to identifying algorithm-based medication-related risks. For example, PDMP dose alerts are based on the widely adopted OME method; however, pharmacists should be aware that OME conversions can be unreliable [39]. For some opioids (e.g., tapentadol), the OME is based on an analgesic effect, but this may not translate directly to the risk of respiratory depression [44]. Similarly, for opioids with complex pharmacology such as methadone, OME conversions can have their limitations [39]. Consequently, using dose alone as a sole risk indicator has resulted in unintended consequences including abrupt tapering, sudden discontinuation or treatment refusal, and patient dismissal [17,45]. Furthermore, there is limited evidence that demonstrates that reducing a patient’s dose reduces their risk, and in fact, reducing or ceasing medications can inadvertently increase risk [46].

Caution should also be exercised in relation to automation bias [14], where pharmacists may attribute greater importance to automations or alerts than other possible risk factors [47] and may result in the dismissal or overlooking of such risks [16]. Results from the current study corroborate that of existing research which suggests caution in using PDMP-related information and alerts alone to make clinical decisions [14]. Best practice guidelines to assist healthcare providers in interpreting, responding to, and communicating PDMP-related information to patients are lacking [48], which poses additional challenges for clinical decision making.

These findings suggest that using a multifaceted approach to risk identification, such as a population-level indicator like a PDMP and a clinical risk screening tool such as the ROOM [25] or the OWLS [27] tools, could better inform the clinical response required. As there is no ‘gold standard’ approach, a multifaceted approach, using a combination of complementary strategies, is recommended [49]. This approach will also provide pharmacists with greater opportunities for assessing a range of risks, both at the point of prescription opioid initiation, and also longitudinally as part of routine and ongoing care. Furthermore, by adopting multiple risk identification approaches, patients may benefit from early identification and intervention, as well as identification of a broader range of risks, both of which result in more informed clinical responses. Knowing the nature of clinical risk may help to determine the course of action while reducing possible unintended consequences that could arise if decisions are primarily driven by alerts alone [16].

As pharmacists are often described as the ‘gatekeepers’ of prescription opioids, they act as the ‘last line of defence’ in the supply chain [50], and are essential to the prevention, management, and treatment of non-medical prescription opioid use [51]. They are responsible for reviewing prescriptions and ensuring they are appropriate and safe to supply [52], and in doing so are required to identify any behaviours suggesting the non-medical use of these medicines. The introduction of policies that outline pharmacists’ roles in identifying and responding to risks may be beneficial. Such policies may also help to ensure that the consistent and regular monitoring of risk becomes part of routine pharmacy practice. Improving support for pharmacists to engage in various risk identification approaches is also important, as is identifying how this support should be provided.

### Strengths and Limitations

When interpreting the results, the following strengths and limitations should be considered. The strengths include the broad sampling of participants from 23 pharmacies from metropolitan, regional, and rural areas across the two most populous Australian states, New South Wales and Victoria. This is the first Australian study to compare at-risk patients according to two well-adopted approaches: PDMP alerts and clinical screening tools. A limitation is that, despite the broad sampling approach, the sample size was relatively small and therefore these results may not be generalisable to all patients who receive opioids, including those on less-regular doses. Specific algorithms from the Victorian PDMP system were used, so different results may be seen with different algorithms utilised in other jurisdictions.

## 5. Conclusions

There are various approaches to assessing opioid-related risk factors, each with its strengths and limitations. The findings have revealed that those identified through PDMP alerts do not correlate well with those identified as having clinical risk using validated screening tools. It is not recommended that pharmacists make clinical decisions based on PDMP alerts alone. The routine use of prescription opioid risk identification approaches such as PDMPs in combination with clinical screening tools will help pharmacists identify a broader range of opioid-related risks. This broader approach may in turn lead to the earlier identification and better clinical management of patients, whilst also mitigating possible harms. 

## Figures and Tables

**Table 1:** Sample characteristics (*n* = 119).

		*n*	%
Demographics			
Gender	Male	39	32.8
	Female	66	55.5
	Unspecified	14	11.8
Age *	18–64 years	65	54.6
	≥65 years	52	43.7
Medications			
Polypharmacy (≥5 medications)	Yes	70	58.8
	No	49	41.2
Prescribed a benzodiazepine or z-drug	Yes	40	33.6
	No	79	66.4
PDMP alerts			
PDMP alert for high dose (>100 OME)	Yes	16	13.4
	No	103	86.6
PDMP alert for medium dose (50–100 OME)	Yes	28	23.5
	No	91	76.5
PDMP alert for high-risk drug combinations **	Yes	5	4.2
	No	114	95.8
Any PDMP alert ^	Yes	46	38.7
	No	73	61.3
ROOM risk			
ROOM severe pain (despite opioid use)	Yes	65	54.6
	No	54	45.4
ROOM opioid use disorder	Yes	37	31.1
	No	82	68.9
ROOM risky alcohol use	Yes	45	37.8
	No	74	62.2
ROOM risk for depression	Yes	27	22.7
	No	92	77.3
ROOM: Any clinical risk #	Yes	94	79.0
	No	25	21.0
OME—Oral Morphine Equivalent			

* Age was unknown for two participants. ** Prescribed either (i) methadone and benzodiazepines or long-acting opioids, or (ii) fentanyl and benzodiazepines or long-acting opioids. ^ PDMP alert for high-dose alert (>100 mg OME) or medium-dose alert (50–100 mg OME) or any high-risk drug combinations. # ROOM risk for severe pain, opioid use disorder, risky alcohol use, or depression.

**Table 2:** Association between prescription drug monitoring program (PDMP) alerts and individual risk indicators of the Routine Opioid Outcome Monitoring (ROOM) tool (*n* = 119).

ROOM Risk Indicator	Any PDMP Alert		X^2^	*p*-Value
	No	Yes		
Opioid Use Disorder (No)	48	34	0.877	0.349
Opioid Use Disorder (Yes)	25	12		
Severe pain (despite opioid use) (No)	35	19	0.502	0.479
Severe pain (despite opioid use) (Yes)	38	27		
Risky alcohol use (No)	44	30	0.293	0.588
Risky alcohol use (Yes)	29	16		
Depression (No)	58	34	0.494	0.482
Depression (Yes)	15	12		
Any ROOM risk (No)	16	9	0.094	0.759
Any ROOM risk (Yes)	57	37		

## Data Availability

Data cannot be shared publicly because participants did not provide consent to their data being made accessible and the ethics approval does not include data sharing.

## Supplementary Materials

The following supporting information can be downloaded at: https://www.mdpi.com/article/10.3390/pharmacy11050164/s1, Figure S1: Prescription drug monitoring program alerts and ROOM risk indicators amongst the whole sample.
