# Supplementary material for: Identifying Prescription-Opioid-Related Risks Using Prescription Drug Monitoring Programs’ Algorithms and Clinical Screening Tools
Source: Pharmacy (Basel). 2023 Oct 13;11(5):164. doi: 10.3390/pharmacy11050164 (PMC10609676; doi:10.3390/pharmacy11050164)
Supplement: Supplementary file 1 [file pharmacy-11-00164-s001.zip › pharmacy-2539700-supplementary.pdf]

Supplementary Figure 1: Prescription drug monitoring program alerts and ROOM risk indicators amongst the whole sample (n=119)*

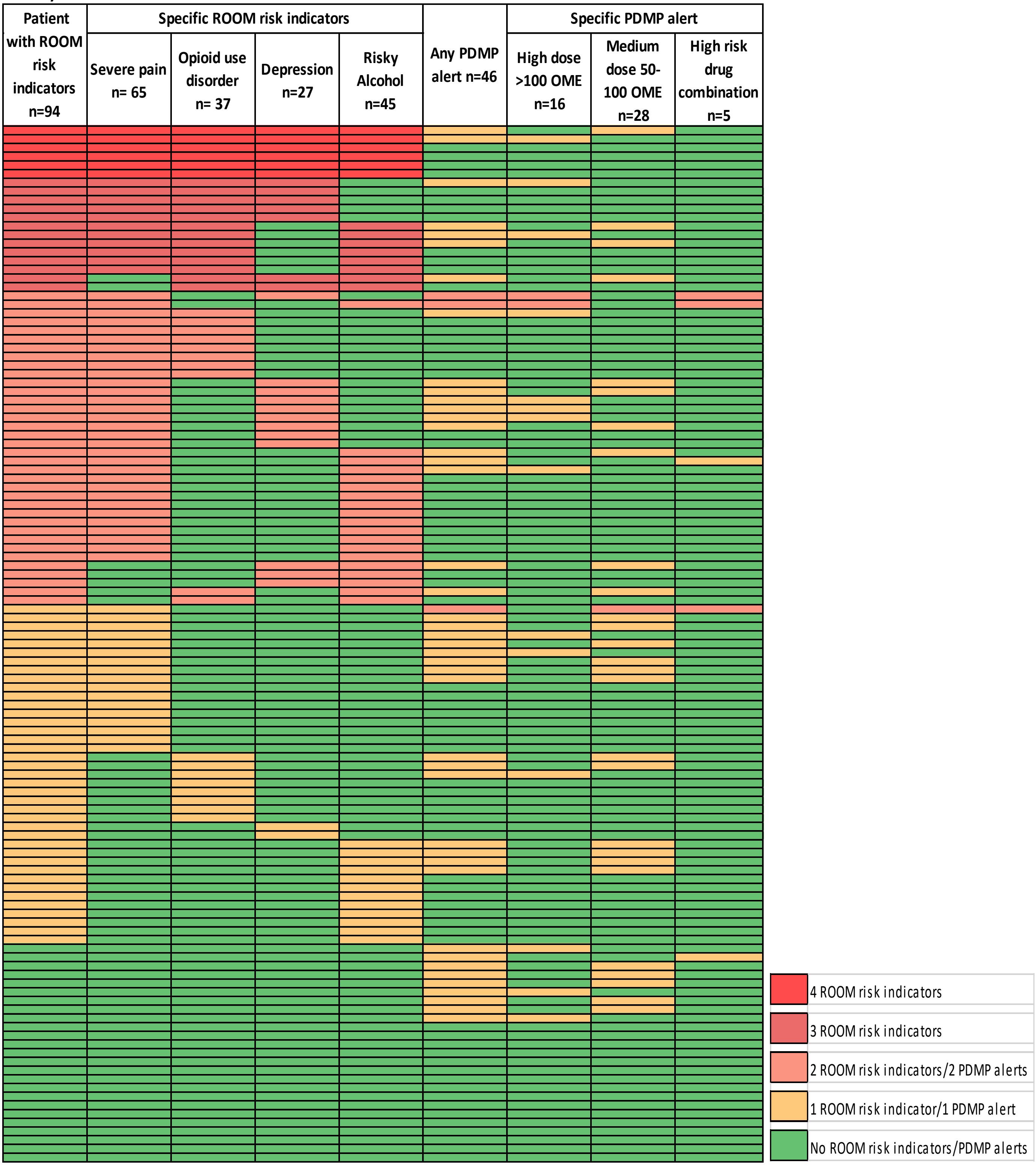

* each line represents an individual patient (n=119)
